# Amphiphilic Chitosan Porous Membranes as Potential Therapeutic Systems with Analgesic Effect for Burn Care

**DOI:** 10.3390/membranes12100973

**Published:** 2022-10-05

**Authors:** Andra-Cristina Enache, Petrisor Samoila, Corneliu Cojocaru, Adrian Bele, Andra-Cristina Bostanaru, Mihai Mares, Valeria Harabagiu

**Affiliations:** 1 Laboratory of Inorganic Polymers, “Petru Poni” Institute of Macromolecular Chemistry, 41A Grigore Ghica Voda Alley, 700487 Iasi, Romania; 2 Laboratory of Antimicrobial Chemotherapy, “Ion Ionescu de la Brad” University of Life Sciences, 8 Mihail Sadoveanu Alley, 700489 Iasi, Romania

**Keywords:** porous membrane, chitosan, lidocaine, burns management, amphiphilic matrix, antibacterial activity

## Abstract

Eliminating or at least lessening the pain is a crucial aspect of burns management, as pain can negatively affect mental health and quality of life, and it can also induce a delay on wound healing. In this context, new amphiphilic chitosan 3D porous membranes were developed and investigated as burns therapeutic systems with analgesic effect for delivery of lidocaine as local anesthetic. The highly porous morphology of the membranes and the structural modifications were evidenced by scanning electron microscopy (SEM), energy dispersive X-ray (EDX) analysis and infrared spectroscopy (FTIR). Improved compression mechanical properties, long-term hydrolytic degradation (28 days) evaluation and high swelling capacities (ranging from 8 to 22.6 g/g) indicate an increased capacity of the prepared membranes to absorb physiological fluids (burns exudate). Lidocaine in vitro release efficiency was favored by the decreased content of cross-linking agent (reaching maximum value of 95.24%) and the kinetic data modeling, indicating that lidocaine release occurs by quasi-Fickian diffusion. In addition to the in vitro evaluation of analgesic effect, lidocaine-loaded chitosan membranes were successfully investigated and proved antibacterial activity against most common pathogens in burns infections: *Staphylococcus aureus* and Methicillin-resistant *Staphylococcus aureus*.

## 1. Introduction

Despite the encouraging progresses made in burn care units in recent decades, burns and scalds still represent a menacing global health issue [1,2]. Every year, approximately 8 million new cases of burns are registered, with a fatality rate around 1:80, according to the World Health Organization data [3] and specialized scientific journals [4,5]. Moreover, burns have a far greater impact than the number of deaths they cause. Therefore, in 2019, more than 7 million disability-adjusted life years (DALYs) were accounted for [4,6], highlighting the consequences of these kind of injuries on the medical, socio-economical and personal life [7].

As one of the most destructive afflictions of the human body [8], burns and their treatment cause one of the most unbearable forms of pain [9]. In light of this, lessening or eliminating pain is a crucial aspect of burns management, especially with it being proved that pain can negatively affect mental health and quality of life and also induce a delay on wound healing [5,10,11,12]. In comparison with infiltration anesthesia and placebos, topical anesthesia offers an effective and safe way to improve the management of pain [13]. In this respect, local anesthetics with prolonged action have been investigated for pain relief in burns, under different formulations, from liposomes and microspheres to micro-needles and solid drug delivery matrices [14]. From a chemical stand point, local anesthetics contain in their structure a hydrophobic aromatic ring, an intermediate chain (either a carboxylic ester or an amide bond) and a hydrophilic tertiary amine [15]. As compared to amino-ester anesthetics, the amino-amides, such as lidocaine, are often preferred in pain management, due to their improved chemical stability, prolonged effect and their metabolic degradation through hepatic oxidation [15,16,17].

Lidocaine (2-(diethylamino)-N-(2,6-dimethyl phenyl)-acetamide) is a widely used amino-amide drug used for numbing a specific area [18,19], being available in various types of formulations (creams, ointments, gels, pastes, sprays and patches) [20,21,22]. In addition to the anesthetic effect, it was demonstrated that lidocaine also possesses potent antioxidant, anti-inflammatory and bactericidal properties, and it can inhibit infiltration and excessive migration of leukocytes [23,24]. However, there are several drawbacks associated with pure lidocaine local administration, such as low solubility in water, risk of systemic absorption and potential toxicity when administered in high single doses [24]. In order to overcome these disadvantages, various drug delivery systems were investigated. Among them, amphiphilic polymeric systems have been shown to be ideal for the solubilization and local release of hydrophobic drugs and also for related toxicity mitigation [25,26].

Natural polysaccharides have been intensively studied in burns management and drug delivery, due to their known biocompatibility, bioactivity, biodegradability and similarities with the extracellular matrix [27,28,29,30]. Chitosan and modified chitosan-based products stand out amongst natural materials used in tissue engineering, given their known biocompatibility, anti-inflammatory, antibacterial, hemostatic and mucoadhesive properties [31,32]. Moreover, chitosan can easily be used in open wounds, improving the functions of inflammatory cells and promoting cell granulation and organization [33]. The ideal structure of chitosan, as a primary derivative of chitin, is based on linear repeating units of β-(1-4)-2-amino-2-deoxy-D-glucopyranose, in which the acetylated units of N-acetyl-D-glucosamine of the chitin are deacetylated to become D-glucosamine units [34]. The functionality of this cationic biopolymer provided by the two hydroxyl groups (at C3 and C6) and one amine group (at C2) allows extensive adjustment of physical, chemical, mechanical and biological properties [35].

Coupled chemical modification of chitosan by acylation with succinic anhydride and crosslinking with an epoxy-terminated disiloxane in order to obtain an amphiphilic matrix was proved in a previous piece of work to be adequate for embedding, and it releases an insoluble antifungal drug [36]. In this context, the present work proposes deeper investigation of this amphiphilic system by obtaining a novel chitosanthree-dimensional porous membrane, to fulfill all the necessary requirements in burns management, such as: (i) pain relief by solubilization and release of lidocaine; (ii) absorption of large amounts of exudate; (iii) maintaining a moist environment needed for wound healing; (iv) offering protection and covering large areas of injured tissue; and (v) possessing antibacterial activity.

## 2. Materials and Methods

### 2.1. Materials

Low molecular weight chitosan (CS) with a degree of deacetylation of 81.6% and an average viscosimetric molecular weight of 290 kDa (based on our previously determinations [37]), lidocaine (LID) of analytical standard, succinic anhydride (SA) and 1,3-Bis(3-glycidyloxypropyl)tetramethyldisiloxane (DS) were provided by Merck Chemical (Saint Louis, MO, USA), while glacial acetic acid (≥99.85%) was purchased from Chemical Company (Iasi, Romania). *Staphylococcus aureus* (ATCC 29213) and MRSA-methicillin-resistant *Staphylococcus aureus* (ATCC 43330) were acquired from American Type Culture Collection (Manassas, VA, USA).

### 2.2. Porous Membranes Preparation

**Figure 1:**
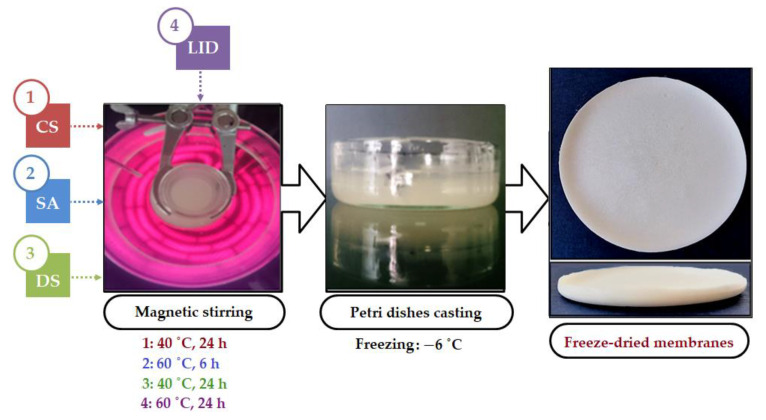
Schematic representation of the preparation process of lidocaine-loaded chitosan membranes.

All the porous membranes were prepared through a sequence of steps, schematically described in Figure 1, culminating in pouring all prepared solutions into Petri dishes (Ø 5 cm) and freezing them at 267 K. Lastly, all the materials were freeze dried (Christ Alpha 3–4 LSCbasic, Osterode, Germany) for 24 h. Therefore, for each membrane, 0.3 g of chitosan was dissolved in 10 mL of aqueous solution of acetic acid 0.5 M, under continuous magnetic stirring (at 313 K, for 24 h). An unmodified chitosan membrane was prepared for analytical purpose (CS-A) by freeze drying the chitosan solution. For the preparation of the chemical-modified membranes, 10 mL of chitosan-acetic acid solution was first subjected to partial N-acylation by addition of 15 mg (or 75 mg, respectively) of succinic anhydride (SA) under stirring at 333 K (6 h). Further, the N-acylated chitosan solution was cross-linked by insertion of 244 mg (or 135 mg, respectively) of epoxy-terminated disiloxane (DS). After pouring in Petri dishes, freezing and freeze-drying, CS-SA/DS-1 and CS-SA/DS-2 membranes were obtained.

For the preparation of the lidocaine (LID)-charged membranes, 0.1 g of LID was added (in situ) over each of CS-SA/DS dispersions (obtained as described in the previous step). The as-obtained LID loaded dispersions were heated at 333 K under magnetic stirring (600 rpm) for 24 h, for the complete dissolution of the drug. After the freeze-drying process, the corresponding LID-charged membranes (CS-SA/DS-1-LID and CS-SA/DS-2-LID) were obtained.

### 2.3. Methods of Characterization

#### 2.3.1. Morpho-Structural Characterization of the Membranes

All the membranes were structurally analyzed using Fourier transform infrared spectroscopy (FTIR—Bruker Vertex 70 spectrophotometer, Ettlingen, Germany) in attenuated total reflectance mode in the wavelength range of 4000–600 cm^−1^ (2 cm^−1^ resolution; 64 scans at room temperature). The morphology of the membranes was investigated at their surface and also in cross-section, using a scanning electron microscope (SEM) with a resolution of 4 nm at 30 kV (FEI QUANTA 200, Brno, Czech Republic). The chemical composition of the membranes was estimated by energy-dispersive X-ray spectrometer (EDX) of the QUANTA 200 system. The diameter of 100 pores was determined based on SEM micrographs using Image J software (version 1.45s, National Institute of Health, Bethesda, Maryland, USA). The collected data were further processed with Minitab 16 Software by elaboration of the pore size distribution histograms and by fitting the experimental data with a LogNormal distribution function. 

#### 2.3.2. Mechanical Properties

The membranes were evaluated for compressive strength using a CETR UMT2 Tribometer (Bruker Corporation, Billerica, MA, USA). Thus, cylindrical samples were cut from each membrane, using a round punch with a diameter of 15 mm, as shown in Figure S1 from Supplementary Materials (SM). The testing procedure is schematically described in Figure S1b (Supplementary Materials). The circular samples were placed on the stationary table of the tribometer and were subjected to compression with an aluminum plate (fixed on the force sensor, through a rigid adapter). An axial displacement of 1 mm in the Z direction of the equipment was established and the force F was monitored throughout the compression process. The compression speed was 0.5 mm/min, and the compressive stress (σ) and strain (ε) were calculated according to Equations (1) and (2), respectively:

(1) $$\sigma \;\left(\mathrm{MPa}\right)=\frac{F}{A},$$

(2) $$\varepsilon \left(\mathrm{mm}/\mathrm{mm}\right)=\frac{\Delta l}{l_{0}},$$

where F is the applied force, A is the cross-sectional area of the sample in compression, l_0_ and l are the initial and deformed sample thickness, respectively, and Δl gives the value of the thickness deformation (Δl = l_0_ − l). The compressive modulus was calculated from the stress–strain curves. For each membrane, 5 different samples were tested and the results were represented as the average of the obtained values.

#### 2.3.3. In vitro Hydrolytic Degradation

The stability of the membranes in physiological fluid was determined according to a method described in the literature [38]. Three samples from each membrane were immersed in PBS solution with a pH of 7.4 and were placed in a shaker-incubator at 310 Kunder stirring (80 rpm). The samples were removed from the incubation medium at certain time intervals (ranging from 1 to 28 days), washed with distilled water and dried at 323 K to constant weight. The degradation of the membranes in neutral pH was evaluated as the weight loss function of time (days), according to Equation (3):

(3) $$D\left(\%\right)=\frac{w_{0}-w_{d}}{w_{0}}\times 100,$$

where D (%) represents the weight loss percentage of the sample, w_0_ corresponds to the initial weight of each sample and w_d_ is the weight of the dried sample at time *t* (days). 

#### 2.3.4. Swelling Behavior and Kinetic Characteristics

Three samples (Ø 15 mm) from each membrane were used to determine the swelling capacity of the membranes (with and without LID). The samples were oven dried prior to the experiments for 1 h at 313 K and weighed. Afterwards, the samples were immersed in 50 mL of phosphate-buffered saline (PBS), maintained at 310 K using a Biosan Orbital Shaker-Incubator (Riga, Latvia). At predetermined time intervals, samples were removed from the immersion medium (excess PBS solution was removed from their surface by blotting with filter paper) and were weighed. The swelling capacities of the porous membranes were determined by the gravimetric method, according to Equation (4), and were graphically represented based on the average of the three determinations, for each membrane:

(4) $$S_{t}\left(g/g\right)=\frac{w_{t}-w_{0}}{w_{0}},$$

where S_t_ (g/g) represents the swelling capacity value at time *t* (min), w_0_ is the initial weight of the sample (dry) and w_t_ is the sample weight at time t after immersion in PBS solution. In order to analyze the swelling properties, the experimental data were fitted by using two mathematical models in SCILAB software (version 6.1.0, ESI Group, Rungis, France). Therefore, the pseudo-second order kinetic model (PSO) was applied to investigate the rate of absorbed PBS solution at equilibrium (Equation (5)) and Korsmeyer–Peppas (K–P) adapted kinetic model was used to determine the water diffusion behavior in the polymeric membranes (Equation (6)) [39,40]:

(5) $$S_{t1}=\frac{k_{s}S_{e1}^{2}t}{1+k_{s}S_{e1}t}\;,$$

(6) $$F=\frac{S_{t2}}{S_{e2}}={\;k}_{p}\times t^{n}\;,$$

where S_t1_ and S_t2_ represent the swelling content of the membranes at time t (min), S_e1_ and S_e2_ are the equilibrium swelling contents, k_s_ is the second-order constant of the swelling rate, F represents the fractional swelling, k_p_ is a constant dependent on the polymeric matrix structure and n is the diffusion exponential coefficient of the PBS solution into the membranes [36,39]. 

#### 2.3.5. In Vitro Lidocaine Release

The in vitro release of lidocaine from the chitosan membranes was evaluated using a NanoDrop ND-1000 spectrophotometer (Marshall Scientific, Cambridge, Massachusetts). The dried samples (323 K to constant weight) were weighed and immersed in 20 mL of PBS solution at 310 K under gentle magnetic stirring (100 rpm). At predetermined time intervals, 20 μL of the initial solution were taken, subsequently replaced with buffer solution and the absorbance values were measured at 264 nm. Based on the obtained values, lidocaine release capacities over time were calculated, according to the calibration curve previously established (Figure S2). The release data were further processed by applying pseudo-first order (PFO) kinetic model, used to describe the diffusional release mechanisms from porous systems (Equation (7)) [41,42]. Additionally, Korsmeyer–Peppas (K–P) model was used to investigate the lidocaine release mechanism, by processing the first 60% of the experimental data, aiming to describe the lidocaine diffusion mechanisms from the chitosan matrix (Equation (8)) [42]:

(7) $$S_{r}=S_{0}\times \left(1-e^{k_{r}t}\right),$$

(8) $$\frac{M_{t}}{{\;M}_{\infty }}={\;k}_{\mathrm{pr}}\times t^{n},$$

where S_0_ is the initial amount of the drug and S_r_ represent the cumulative amount of the drug release at time t, k_r_ is the pseudo-first order release constant, M_t_/M_∞_ represents the drug release fraction at time t (M_t_ and M_∞_ being the amount of drug released at time t and at infinite time, respectively), k_pr_ is the kinetic K–P constant and n is the characteristic diffusion (release) coefficient, providing information about the type of the release mechanism (n < 0.5—quasi-Fickian diffusion; n = 0.5—unidirectional/Fickian diffusion; 0.5 < n < 1—non-Fickian transport; n = 1—Case II non-Fickian transport; n > 1—non-Fickian supercase II transport [43].

#### 2.3.6. Antibacterial Activity

The antibacterial activity of unmodified chitosan and lidocaine-charged chitosan membranes was evaluated on two strains of *Staphylococcus aureus*: *Staphylococcus aureus* and methicillin-resistant *Staphylococcus aureus* (MRSA). All microorganisms were stored at 193 K in 10% glycerol. Microbial suspensions were prepared in sterile saline in order to achieve turbidity, optically comparable to that of 0.5 McFarland standards (containing approximately 1 × 108 CFU mL^−1^ for all bacteria). A volume of 0.2 mL of each inoculum was spread on Petri dishes (with a pre-poured Mueller–Hinton agar culture medium). After drying, lidocaine-charged chitosan samples were added, measuring the zone of growth inhibition under standard conditions after 24 h of incubation at 309 K. For analytical purpose, unmodified chitosan membrane was also tested. All the measurements were realized on three different samples for each membrane. The diameter of the inhibition zones around the samples was measured using an electronic caliper with a digital display. 

## 3. Results and Discussions

### 3.1. Porous Membrane Preparation

Knowing that the commercially available patches hinder the healing process because of their adhesion properties and also inhibit the hydration of the wounds [22], lidocaine-charged chitosan porous membranes were designed to combat these drawbacks by partial modification of the amino groups of chitosan (CS) with succinic anhydride (SA) and the cross-linking of succinyl-chitosan derivative with 1,3-bis-(3-glycidyloxypropyl)-1,1,3,3-tetramethyldisiloxane (DS), as schematically represented in Figure S3. Among the main chitosan modifications investigated in recent years (i.e., acylation, alkylation, thiolation, carboxymethylation, quaternization, nitration, phosphorylation and sulfation) [44], N-acylation of chitosan with succinic anhydride was investigated for different wound dressing, cosmetic materials and drug delivery systems, mainly due to the unique properties conferred by the presence of carboxylic groups [45]. The increase in the chitosan solubility in physiological pH and the possibility of an increase in the absorption capacities of large amounts of physiological fluids constitutes an advantage for the burns management applications. In addition, the use of the epoxy compounds, such as DS, allows the attaching of alkyl fragments to the hydrophilic chitosan derivative, providing an amphiphilic character to the final material. This fact might be essential for the stabilization and release of insoluble drugs with hydrophobic sequences in their structure (such as lidocaine) [46]. Moreover, the DS crosslinking agent can increase the hydrodynamic and mechanical stability of the membranes formulations and can also regulate the tissue adhesion by varying the hydrophobic/hydrophilic ratio.

A similar modified chitosan system (in the form of hydrogels and thin films formulations) was investigated by our group in a previous piece of work for the embedment and release of the insoluble antifungal drug nystatin [36]. In this work, a new approach in the preparation of the chitosan formulation is pursued to fulfill the burns therapeutic system requirements. The chemical composition of the lidocaine-charged membranes (CS-SA/DS-1-LID and CS-SA/DS-2-LID) is detailed in Table 1. For the comparative investigation of the physical, chemical and mechanical properties, non-charged membranes with the same chemical composition (CS-SA/DS-1 and CS-SA/DS-2) and unmodified chitosan membrane (CS-A) were prepared (Table 1). In the preparation process, lidocaine (LID) was added in situ, following the chemical modification of chitosan (as described in Section 2.2), using a CS/LID weight ratio of 3:1. This drug dispersion method was chosen to achieve a uniform dispersion of lidocaine into the chitosan matrix, required for precise administration and facile delivery of the anesthetic active principle [32]. Moreover, the amount of LID was also related to the size of the final membrane (Ø 5 cm), as it was proved that applying 5 g of the commercial product EMLA cream (containing 0.125 g lidocaine and 0.125 g prilocaine) on 25 cm^2^ burn surfaces is far from reaching the level of toxicity [47]. In addition, all the chitosan-based membranes were obtained by the freeze-drying method to induce additional advantages to the chitosan therapeutic system, such as microporosity, high absorption capacity of the exudate, wound protection and ability of covering large injured surfaces, and tissue regeneration due to hydrophilic three-dimensional polymeric network [27].

### 3.2. Structural Characterization of the Membranes

**Figure 2:**
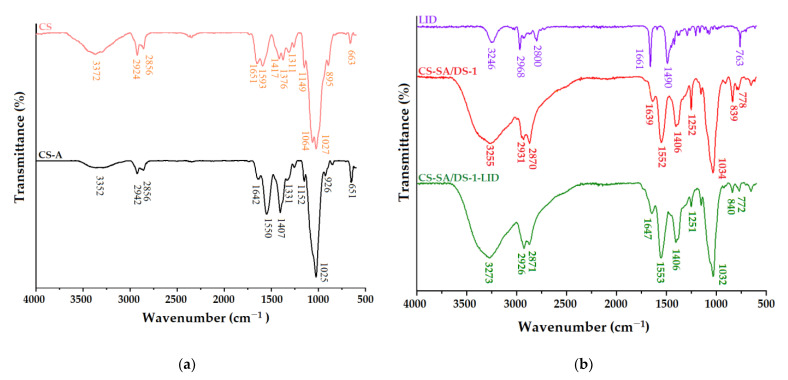
FTIR spectra of: (**a**) pristine chitosan (CS) and chitosan membrane (CS-A), obtained after CS dispersion in 0.5 M acetic acid solution; (**b**) pure lidocaine (LID), non-charged chitosan membrane (CS-SA/DS-1) and lidocaine-charged chitosan membrane (CS-SA/DS-1-LID).

Dissolving chitosan in acidic pH (0.5 M acetic acid solution) led to partial protonation of the amino groups, as confirmed by FTIR infrared spectroscopy. In Figure 2a, the chitosan membrane (CS-A) spectrum is compared to pure chitosan spectrum (CS). The latter spectrum shows the polysaccharide characteristic bands, discussed in detail in a previous piece of work [37]. Briefly, we can observe the specific chitosan absorption bands at 1651 cm^−1^ (C = O bond of amide I), 1593 cm^−1^ (in-plane deformation vibration of the NH_2_ primary amino group and the amide II stretching vibrations) and 1311 cm^−1^ (C–N bond (amide III) stretching vibrations); the 1149 cm^−1^ absorption band is given by the asymmetric stretching vibrations of glycoside C–O–C bonds, as are 1064 and 1027 cm^−1^ (stretching vibrations of C–O bond, specific for the polysaccharides structure) [37]. On the other hand, the CS-A chitosan membrane spectrum reveals the shift of the band corresponding to amide I from 1651 to 1642 cm^−1^ and the appearance of two new absorption bands at 1550 and 1407 cm^−1^. These spectral changes are a consequence of the ionization of the amino groups of chitosan (NH_3_^+^) and the Coulomb interactions that occur with the carboxylate groups. At the same time, the reduction in intensity of the absorption band in the range 3400–3100 cm^−1^ confirms the conversion of NH_2_ groups into NH_3_^+^ groups by protonation. In addition, the band at 651 cm^−1^ corresponds to the O–C–O (δ) vibrational mode of acetic acid. All the differences detected between the two FTIR spectra indicated the protonation of the polysaccharide NH_2_ groups by H^+^ ions provided by acetic acid [48,49].

The comparison between the spectra of lidocaine-charged (CS-SA/DS-1-LID) and non-charged (CS-SA/DS-1) chitosan membranes is realized in Figure 2b. Likewise, the FTIR spectrum of lidocaine is depicted in Figure 2b, while the spectra of the SA and DS were represented and discussed in the Supplementary Material (Figure S4). From the lidocaine spectrum one may observe that the main absorption bands can be identified as follows: stretching vibrations of the N – H group appears at 3246 cm^−1^, C = O bond (amide I) stretching vibrations can be observed at 1661 cm^−1^ and the C – N group (amide III) gives an absorption band at 1490 cm^−1^. The asymmetric and symmetric stretching vibrations of the CH_3_ group appear at 2966 cm^−1^ and 2800 cm^−1^ [50]. 

The chitosan membrane without lidocaine (CS-SA/DS-1) spectrum shows absorption bands in accordance with CS chemical modifications. The succinylation of the polysaccharide induced the disappearance of the 3016 cm^−1^, 1861 cm^−1^ and 1773 cm^−1^ absorption bands (from the SA spectrum—Figure S4), following the opening of the anhydride cycle [36]. In addition, the increase in intensity and slight displacement of primary N – H absorption band indicates that the succinylation reaction occurred at the free NH_2_ group of CS with the formation of new amide units – NH – CO – [51]. The crosslinking process with DS was also confirmed by the disappearance of the epoxy C – H group absorption band from 3055 cm^−1^ (shown in Figure S4) and the appearance of new siloxane (Si – CH_3_) specific absorption bands at 1252 cm^−1^, 839 cm^−1^ and−778 cm^−1^ [36].

The introduction of lidocaine into the CS-SA/DS-1 membrane, to obtain the CS-SA/DS-1-LID sample, caused an increase in the intensity of the absorption band corresponding to the asymmetric stretching vibrations of C – H and the appearance of the primary amide absorption band at 1647 cm^−1^, due to the overlap of the amide I band from CS with the one from LID structure. Due to the structural similarities of the polysaccharide with lidocaine, no other significant differences were found comparing the spectra of the lidocaine-charged membrane with the non-charged membrane.

### 3.3. Membranes Morphological Evaluation

**Figure 3:**
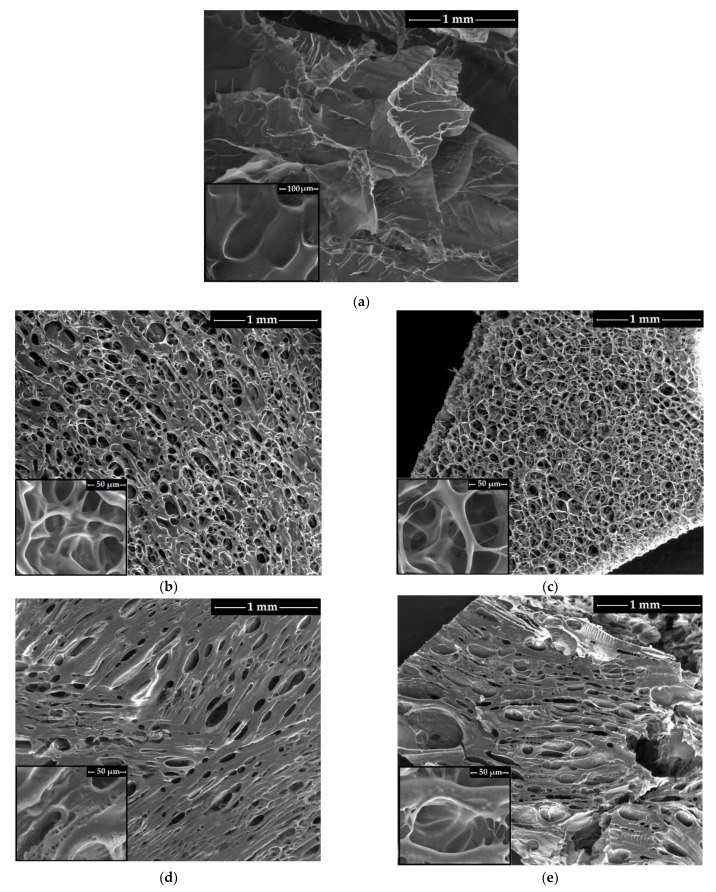
SEM images on the surface of non-charged chitosan membranes: (**a**) CS-A, (**b**) CS-SA/DS-1 and (**c**) CS-SA/DS-2 and of the lidocaine-charged membranes: (**d**) CS-SA/DS-1-LID and (**e**) CS-SA/DS-2-LID.

The morphological analysis of all the chitosan membranes was carried out by scanning electron microscopy, both on the surface of the membranes (Figure 3) and on their cross-section (Figure S5). All the membranes presented a porous tridimensional structure, as a result of the freeze-drying process. However, the membranes morphology also varied according to their composition, which was qualitatively estimated by EDX and described in Figure S6. 

The unmodified chitosan membrane reveals a specific phase-segregated surface morphology due to the partial crystallinity of the polymer (Figure 3a). Moreover, the layered cross-section structure (Figure S5a), with large pores oriented in the same direction, is specific to the freeze-dried chitosan samples [52]. This morphology is explained by the fact that pre-freezing the chitosan solutions at 267 K led to a slower cooling rate, allowing the ice crystal to grow inside the material. The opposite behavior was observed by others when freezing the CS samples at 77 K [53].

The chemical modification of chitosan by succinylation with SA and cross-linking with DS induced a surface superporous morphology, with rounded, well-defined and irregular sized pores (Figure 3b,c). The introduction of lidocaine into the chemically modified chitosan system induced a surface structural reorganization (Figure 3d,e) by increased matrix density, as a result of the hydrophobic groups from the lidocaine structure. The observed decrease in the pore frequency corroborated that the thickened pores walls can be explained by electrostatic repulsion between the lidocaine hydrophobic group and disiloxane hydrophobic sequences [54]. On the other hand, one may observe that surface morphology of modified chitosan membranes (charged and non-charged with LID) depend on the cross-linker concentration. Therefore, the CS-SA/DS-1 and CS-SA/DS-1-LID membranes with higher content of DS cross-linking agent (Figure 3b and 3d) presented a thickening of the surface network walls, as compared to the CS-SA/DS-2 and CS-SA/DS-2-LID membranes (Figure 3c,e). Moreover, in Figure 3d (insert) a segregation can be observed, as a result of the incompatibility between the DS and LID hydrophobic groups and the succinylated CS hydrophilic ones.

For all the modified chitosan membranes, the surface pores are interconnected and form channels to and/or from the inner structure, which also presented a superporous morphology with irregular shaped pores with relatively thin walls (Figure S5b,c). The lidocaine-charged membranes tridimensional porous morphology is suitable for therapeutic applications, allowing the facile penetration of physiological fluids and the diffusion of drug molecules. At the same time, the presence of rougher walls should facilitate the cell adhesion and proliferation [55].

### 3.4. Pore Size Distribution

**Figure 4:**
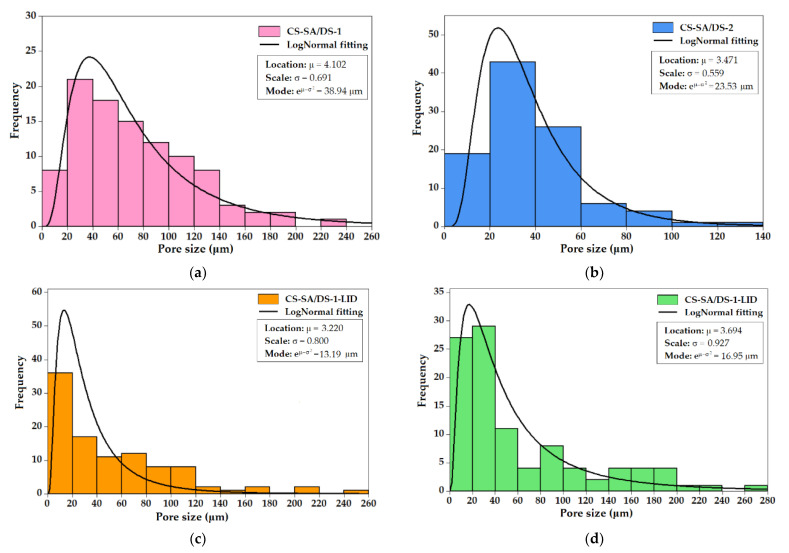
Histograms of pore distribution at the surface of: non-charged CS membranes (**a**) CS-SA/DS-1 and (**b**) CS-SA/DS-2; lidocaine-charged membranes (**c**) CS-SA/DS-1-LID and (**d**) CS-SA/DS-2-LID; the experimental data were fitted with LogNormal distribution function using Minitab 16 Software.

Based on the SEM micrographs, statistical analysis of the pore size was performed for all the modified CS membranes. Histograms of pore-size distribution on membranes surface and cross-section are given in Figure 4 and Figure S7, respectively. According to Figure 4, all the membranes presented a right skewed type of distribution of the pore size, which is characterized by a LogNormal distribution (continuous probability distribution applied to the pores sizes ranging from higher number of small pores to fewer larger pores) [56]. The location (μ) and scaling (σ) parameters were used to determine the mode of the LogNormal distribution, as it represents the pore size values with highest probability of occurrence (between 13 and 39 μm).

The non-charged chitosan membranes (Figure 4a,b) are characterized by pores with dimensions ranging from 7 to 225 μm (CS-SA/DS-1) and from 6 to 132 μm (CS-SA/DS-2), respectively. It can be noticed that the higher amount of cross-linking DS agent induced an increased number of larger pores in CS-SA/DS-1 membrane, as compared with CS-SA/DS-2, by increasing the CS matrix density. Moreover, the introduction of the lidocaine in the CS system led to the appearance of even larger pores (up to 241 μm for CS-SA/DS-1-LID and up to 276 μm for CS-SA/DS-2-LID, respectively), as shown in Figure 4c,d, due to polymer chain entanglements. A similar trend was also observed in the pore distribution histograms in the membrane cross-sections, as shown in Figure S7a–d.

It should be mentioned that, in the case of CS-SA/DS-1-LID porous membrane (Figure 4c), the higher pore count frequency was recorded in the range of 2–20 μm, explained by the phase segregation (due to large amount of DS) and the appearance of a large number of micro-pores ≤ 10 μm (as shown in the insert from Figure 3d). The increased porosity observed in the SEM images (Figure 3) and the pore sizes, mainly in the range of 10–100 μm, suggest that the membranes are suitable for therapeutic drug delivery systems, either as drug absorption and release membranes or as matrices for cell growth.

### 3.5. Mechanical Properties

Lidocaine- charged porous membranes (CS-SA/DS-1-LID and CS-SA/DS-2-LID) were evaluated in terms of mechanical properties by reference to the unmodified chitosan membrane (CS-A) and the non-charged corresponding membranes ((CS-SA/DS-1 and CS-SA/DS-2, respectively). Based on the compression stress–strain curves, the values of the compression strength and the mean compression modulus were determined and reported in Table 2.

The unmodified chitosan membrane (CS-A) had higher values of compression strength and modulus, as compared to CS-SA/DS-1 and CS-SA/DS-2 membranes. This fact can be explained by the specific superporous morphology of these samples (as highlighted in Section 3.3). By contrast, the porous membranes loaded with lidocaine have a special behavior, which can also be correlated with their morphology. CS-SA/DS-1-LID and CS-SA/DS-2-LID showed higher values compared to those of the uncharged membranes, mainly due to the crystal structure of LID and the non-covalent interactions (hydrophobic and hydrogen bonds) that occur between LID molecules [57] or between LID and the polymeric matrix [58].

Membranes with a higher cross-linker concentration (CS-SA/DS-1 and CS-SA/DS-1-LID) exhibited increased compression strength, as compared to the membranes with lower amounts of DS (CS-SA/DS-2 and CS-SA/DS-2-LID). It can be stated that the thickening of the pore walls and the increase in the density of the CS matrix cause an increase in the compressive strength. Moreover, the compression modulus also decreased with decreasing cross-linker (DS) content from 0.17 to 0.13 MPa for non-charged membranes (CS-AS/DS-1 and CS-AS/DS-2, respectively) and from 0.48 to 0.32 MPa for lidocaine-charged membranes (CS-SA/DS-1-LID and CS-SA/DS-2-LID, respectively). These findings are in close agreement with the literature [59] and occur due to the cross-linking reactions between the amino groups of chitosan and the terminal epoxy groups of DS.

### 3.6. In Vitro Hydrolytic Degradation

Porous membranes suitable for tissue regeneration must have degradation capacities proportional to the rate of tissue regeneration. The determination of weight loss over time can be a direct measure to quantify the degradation of the polysaccharide. Thus, the hydrolytic degradation behavior of the lidocaine-charged chitosan porous membranes was investigated by comparison with the non-charged membranes. In this respect, the samples were immersed in simulated physiological fluid (PBS) for 28 days, as the necessary time of healing acute wounds and burns of I and II degree [60].

**Figure 5:**
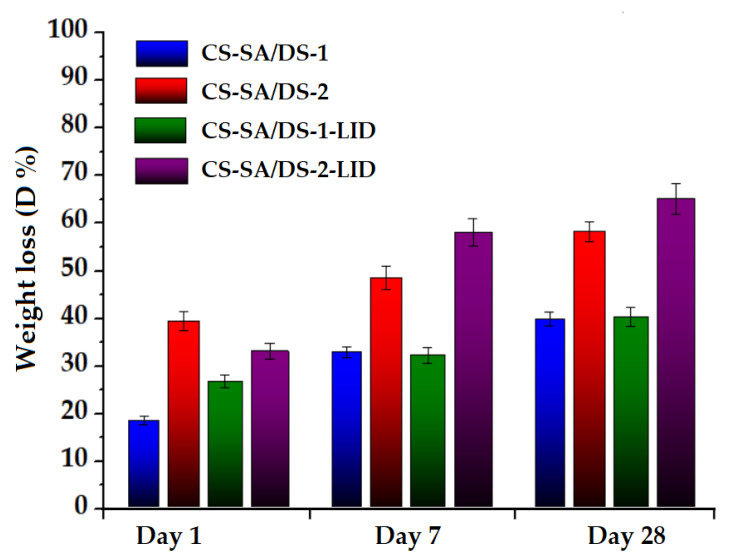
Weight losses of the chemically modified chitosan membrane with and without lidocaine content.

The weight loss of the chemically modified chitosan membranes, after immersion in PBS solution at 310 K, is graphically represented in Figure 5. From Figure 5 data, one may observe that all the membranes presented a rapid mass loss in the first 24 h (around 18% for CS-SA/DS-1, 39% for CS-SA/DS-2, 27% for CS-SA/DS-2 and 33% for CS-SA/DS-2, respectively), followed by a slower degradation rate in the next 27 days. As expected, increasing the amount of succinic anhydride and decreasing the amount of siloxane compound induced an increase in membranes weight loss during the entire experimental period. Thus, after 28 days of experiment, CS-SA/DS-2 and CS-SA/DS-2-LID presented weight losses increased by about 18% and 25%, as compared with their corresponding strongly cross-linked membranes (CS-SA/DS-1 and CS-SA/DS-1-LID). This trend is dependent on the membranes morphology and mechanical properties previously discussed.

At the same time, an increase in the degradation rate of lidocaine-charged membranes was noted, in contrast with the non-charged membranes, CS-SA/DS-2-LID registering the highest weight loss of 65%.

### 3.7. Swelling Behavior and Kinetic Properties

**Figure 6:**
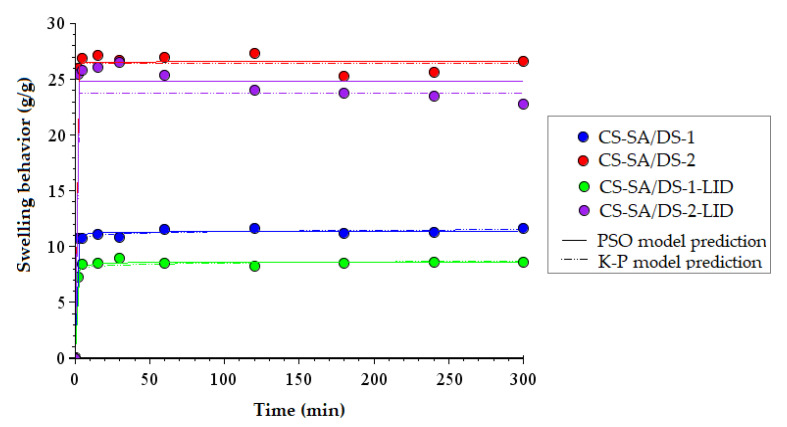
Swelling capacity of lidocaine-charged (CS-SA/DS-1-LID and CS-SA/DS-2-LID) and non-charged (CS-SA/DS-1 and CS-SA/DS-2) porous chitosan membranes, evaluated in PBS solution at 310 K. Experimental data were fitted with Pseudo-second order (PSO) and Korsmeyer–Peppas (K–P) kinetic models.

The capacity of the chitosan membranes to absorb physiological fluids, such as burns exudates, plays a crucial role in maintaining a moist environment over burns and also influences the drug release efficiency [55]. The swelling capacity of the chitosan-based porous membranes (charged and non-charged) was evaluated in simulated conditions (in PBS solution at 310 K), and the experimental data were displayed in Figure 6. One may see that all the membranes exhibited an accelerated swelling capacity (after only 2 min), reaching the almost maximum swelling capacity values within the first 30 min after immersion in PBS. As expected, maximum swelling capacities of 26.63 g/g and 22.80 g/g (Table S1) were achieved after 5 h for the non-charged CS-SA/DS-2 membrane and lidocaine-charged CS-SA/DS-2-LID membrane, respectively. These results are in accordance with the specialized literature and are explained by the higher content of SA increasing the presence of –COOH groups into the system. Thus, the hydrophilic and amphoteric properties, necessary for the diffusion of water in the polymeric matrix are ensured [54]. On the other hand, increasing the content of DS units has the opposite effect, by inhibiting the swelling of membranes in PBS, both by increasing the crosslinking density, as well as the hydrophobic character [59].

By comparison with the non-charged membranes, one may observe that lidocaine-charged membranes with the same chemical composition presented slightly reduced swelling capacities (with 3–4 g/g). This might be explained by increased hydrophobicity due to the presence of hydrophobic groups in the lidocaine structure and also by the morphology of LID-charged membranes (see Figure 3d,e), with increased matrix density.

The rate of absorbed PBS solution at equilibrium and water diffusion mechanism into the polymeric membranes were investigated by processing the experimental data with pseudo-second order (PSO) and Korsmeyer–Peppas (K–P) kinetic models, as represented in Figure 6, and the corresponding kinetic parameters are detailed in Table S1. One may notice that predicted values of the swelling capacity at equilibrium (S_e1_) after 5 h are in line with the experimental values. In addition, the diffusion coeficient (n) determined from the K–P equation (Equation (6)) had values smaller than 0.5, suggesting a Fickian diffusion mechanism, characterized by the rapid diffusion of water molecules into the polymeric porous membrane (compared to the relaxation of the polysaccharide chains) [39,40].

The swelling capacity determinations are consistent with the results obtained in the degradation assessment (Section 3.6). The membranes with higher swelling capacities (CS-SA/DS-2 and CS-SA/DS-2-LID) presented the highest weight losses, mainly due to the fact that hydrolysis is accelerated by a greater water uptake [38]. However, the increased absorption capacities of the PBS solution recommend these membranes for their use in the treatment of burn injuries, which produce a larger amount of exudate, with the possibility of covering large areas of injured tissue.

### 3.8. In Vitro Release of Lidocaine

**Figure 7:**
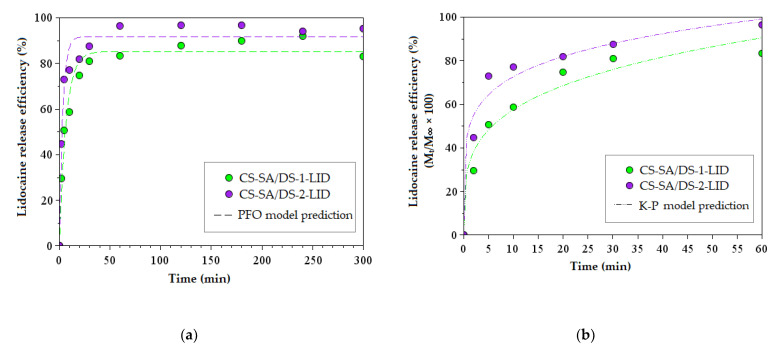
In vitro lidocaine release from chitosan porous membranes and experimental data processing by: (**a**) fitting by pseudo-first order (PFO) kinetic model; (**b**) fitting by Korsmeyer–Peppas (K–P) kinetic model (with the processing of the first 60% of data).

In vitro lidocaine release from the porous chitosan membranes was performed in PBS solution at 310 K. LID release efficiency as a function of time was graphically represented in Figure 7a and its values after 5 h were reported in Table S2. According to Figure 7a, it can be noticed that LID release from CS-SA/DS-2-LID membrane (95.24 %) is superior to the release from CS-SA/DS-1-LID membrane (83.16%). Lidocaine release is favored by the increased amount of SA and decreased content of DS cross-linker, and the results are in accordance with the swelling behavior of the both membranes. Thus, in both cases, a two-step release was observed, with an initial rapid release in the first 20 min (around 74 and 81% from the CS-SA/DS-1-LID and CS-SA/DS-2-LID membranes, respectively. Further, the process continued slowly, reaching maximum values of the LID release after approximately 1 h and slightly changes after long contact times (5 h). From a clinical point of view, an initial rapid release of lidocaine in the early stage is beneficial, as it helps one to reach a therapeutic concentration of the drug in the shortest time, and the subsequent sustained release helps to maintain a minimum effective concentration [61]. It should be noted that the porous morphology of the membranes induced by the freeze-drying process plays an important role in lidocaine release, by significantly increasing the drug release efficiency, compared to chemically similar membranes obtained by slow solvent evaporation [36].

The release experimental data were further processed by PFO (Figure 7a) and K–P (Figure 7b) models and the kinetic parameters were presented in Table S2. The PFO kinetic model provided a good estimation of the lidocaine release efficiency, showing that the predicted values are in accordance with the experimental ones. In addition, the n coefficient determined from the K–P fitting has values of 0.25 and 0.17 (for CS-SA/DS-1-LID and CS-SA/DS-2-LID, respectively), indicating that lidocaine release occurs by quasi-Fickian diffusion [43].

### 3.9. Antibacterial

**Figure 8:**
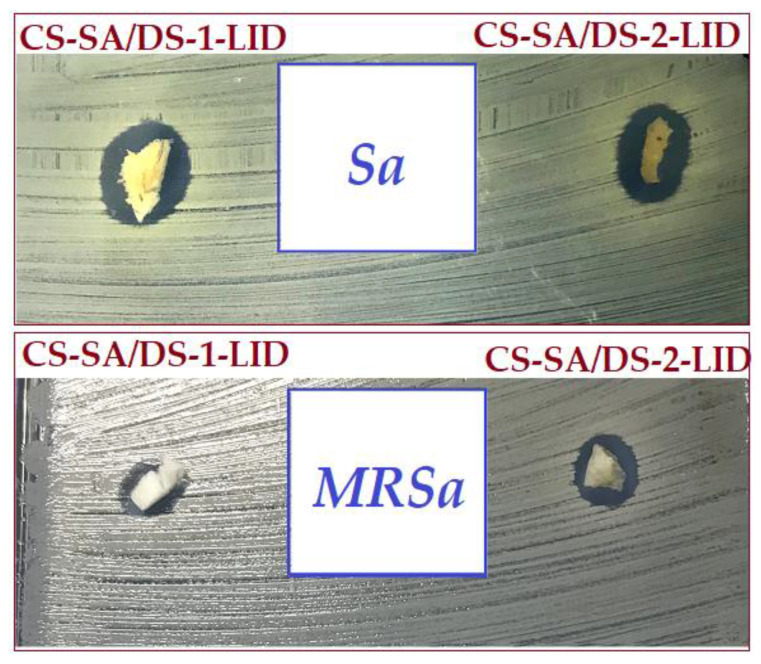
Antibacterial activity of lidocaine-charged porous membranes against *Staphylococcus aureus* (*Sa*) and Methicillin-resistant *Staphylococcus aureus* (*MRSa*).

Burn wounds are a favorable place for the development of bacterial infections. *Staphylococcus aureus* and Methicillin-resistant *Staphylococcus aureus* (MRSa) are the most common pathogens, the latter being well-known for invasive infections [62,63]. In this context, the antibacterial effect of the lidocaine-charged porous membranes was evaluated against both Gram-positive *Staphylococcus* strains (Figure 8). With values of the inhibition zone diameters ranging between 8 and 11 mm, both lidocaine-loaded chitosan membranes showed a significant inhibitory effect for the analyzed strains, mainly due to the presence of negative carboxylic groups, resulting from the modification of chitosan with succinic anhydride. The synergistic contribution of lidocaine, which in addition to its anesthetic effect is known to have also antibacterial effect, cannot be neglected either [64].

## 4. Conclusions

Amphiphilic chitosan-based membranes were prepared by coupled chemical modification of the polysaccharide by acylation with succinic anhydride and cross-linking with 1,3-Bis(3-glycidyloxypropyl)tetramethyldisiloxane at two different concentrations. The lidocaine, an anesthetic drug, was uniformly dispersed into the chitosan matrix by adding it in situ during the preparation process of membranes. For the sake of comparison, non-charged membranes were also studied.

Three-dimensional systems with increased microporosity were obtained by using the freeze-drying method, and their morphology was proved depending on the cross-linker concentration. The increased porosity observed from the SEM micrographs and the pores usually being sized between 10–100 μm indicated the suitability of the membranes for therapeutic drug delivery systems. The lidocaine-charged membranes showed improved mechanical properties, as compared with the non-charged samples, with higher values of compression strength and of mean compression modulus values.

In vitro hydrolytic degradation was long-term evaluated, revealing weight losses of 40–65% after 28 days for the lidocaine-containing membranes. The membrane with a lower concentration of cross-linking agent presented higher swelling capacity (22.8 g/g after 5 h), by comparison with the membrane with higher amount of disiloxane cross-linker (8.6 g/g), recommending these materials in the treatment of burn injuries, which produce a larger amount of exudate.

In vitro lidocaine release disclosed an initial rapid release in the first 20 min (releasing 74 and 81% depending on cross-linker concentration) which is beneficial for the pain relief. Moreover, the processing of the experimental data indicated a quasi-Fickian diffusion mechanism of lidocaine release from the polymeric matrix. Additionally, to induce an analgesic effect, lidocaine-loaded chitosan membranes showed a significant inhibitory effect against *Staphylococcus aureus* and Methicillin-resistant *Staphylococcus aureus* (MRSa), which are responsible for the most burns bacterial infections.

However, the design and investigation of these therapeutic systems with dual effect (analgesic and antibacterial) remains a challenge and of great interest, which is why we propose future research directions regarding the optimization of porous membranes in terms of: system stability under realistic conditions, lidocaine leaching properties and determination of the storage temperature of the system, as well as biocompatibility testing and clinical evaluation of the membranes.

## Figures and Tables

**Table 1:** Composition of chitosan-based membranes.

Hydrogel/Film ^1^ Code	CS (g)	SA ^2^ (mg)	DS ^2^ (mg)	LID (g)
CS-A	0.3	-	-	
CS-SA/DS-1 CS-SA/DS-2	0.3	-	-	
CS-SA/DS-1-LID	0.3	15	244	0.1
CS-SA/DS-2-LID	0.3	75	135	0.1

**Table 2:** Mechanical properties of the chitosan membranes.

Membrane	Maximum Force (N)	Compression Strength (MPa)	Mean Compression Modulus (MPa)
CS-A	3.05 ± 0.51	0.06 ± 0.01	0.31 ± 0.05
CS-SA/DS-1	1.73 ± 0.29	0.03 ± 0.01	0.17 ± 0.04
CS-SA/DS-2	1.27 ± 0.27	0.02 ± 0.01	0.13 ± 0.03
CS-SA/DS-1-LID	4.62 ± 1.60	0.09 ± 0.03	0.48 ± 0.16
CS-SA/DS-2-LID	2.86 ± 1.16	0.04 ±0.02	0.32 ± 0.12

## Data Availability

Not applicable.

## Supplementary Materials

The following supporting information can be downloaded at: https://www.mdpi.com/article/10.3390/membranes12100973/s1, Figure S1: Mechanical properties testing: (a) 15 mm diameter sample from CS porous membrane; (b) Schematic representation of the compressive strength testing device; Figure S2: Calibration curve of lidocaine, at a wavelength of 264 nm, in the concentration range of 0–2 mg/mL; Figure S3: Schematic representation of the amphiphilic chitosan system (obtained by functionalization with succinic anhydride (SA) and cross-linking with 1,3-Bis(3-glycidyloxypropyl)tetramethyldisiloxane (DS)) for the embedment of insoluble lidocaine drug; Figure S4: FTIR spectra of succinic anhydride (SA) and 1,3-bis-(3-glycidyloxypropyl) -1,1,3,3-tetramethyldisiloxane (DS); Figure S5: SEM images on the cross-section of non-charged chitosan membranes: (a) CS-A, (b) CS-SA/DS-1 and (c) CS-SA/DS-2, and of the lidocaine-charged membranes: (d) CS-SA/DS-1-LID and (e) CS-SA/DS-2-LID; Figure S6: Structural elements determined by EDX in the section of: (a) CS-A unmodified membrane; (b) CS-SA/DS-1 modified membrane; and (c) CS-SA/DS-1-LID lidocaine-loaded chitosan membranes. Besides the specific C, O and N atoms of the chitosan and lidocaine compounds, the presence of Si atoms was noticed as the result of the cross-linking process; Figure S7: Histograms of pore distribution in the cross-section of: non-charged CS membranes (a) CS-SA/DS-1 and (b) CS-SA/DS-2; lidocaine-charged membranes (c) CS-SA/DS-1-LID and (d) CS-SA/DS-2-LID; the experimental data were fitted with LogNormal distribution function using Minitab 16 Software; Table S1: Swelling kinetic models parameters (PSO—pseudo-second order; K-P—Korsmeyer-Peppas) and maximum swelling capacity values (g/g) of the porous membranes, after 5 h of immersion in PBS solution; Table S2: Release kinetic models (PFO—pseudo-first order and K-P—Korsmeyer-Peppas) parameters 1. Lidocaine release efficiency (%) from chitosan porous membranes after 5 h.
