# Supplementary material for: Amphiphilic Chitosan Porous Membranes as Potential Therapeutic Systems with Analgesic Effect for Burn Care
Source: Membranes (Basel). 2022 Oct 5;12(10):973. doi: 10.3390/membranes12100973 (PMC9611202; doi:10.3390/membranes12100973)
Supplement: Supplementary file 1 [file membranes-12-00973-s001.zip › membranes-1947712-supplementary.pdf]

membranes

Supplementary materials

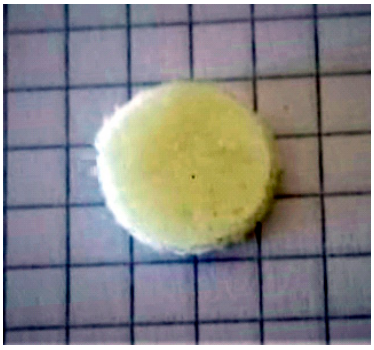

(a)

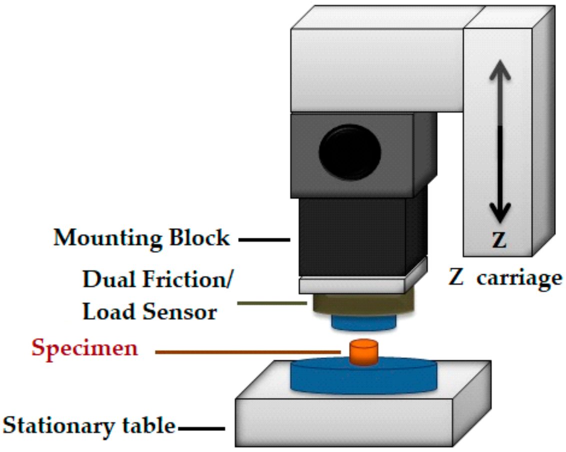

(b)

Figure S1. Mechanical properties testing: (**a**) 15 mm diameter sample from CS porous membrane; (**b**) Schematic representation of the compressive strength testing device.

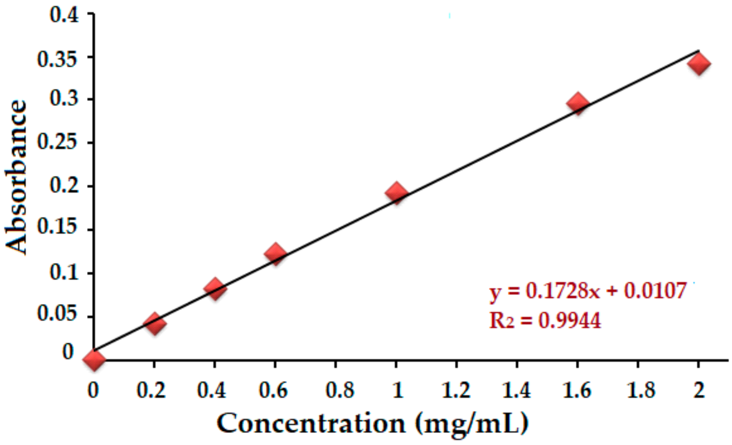

Figure S2. Calibration curve of lidocaine, at a wavelength of 264 nm, in the concentration range of 0-2 mg/mL.

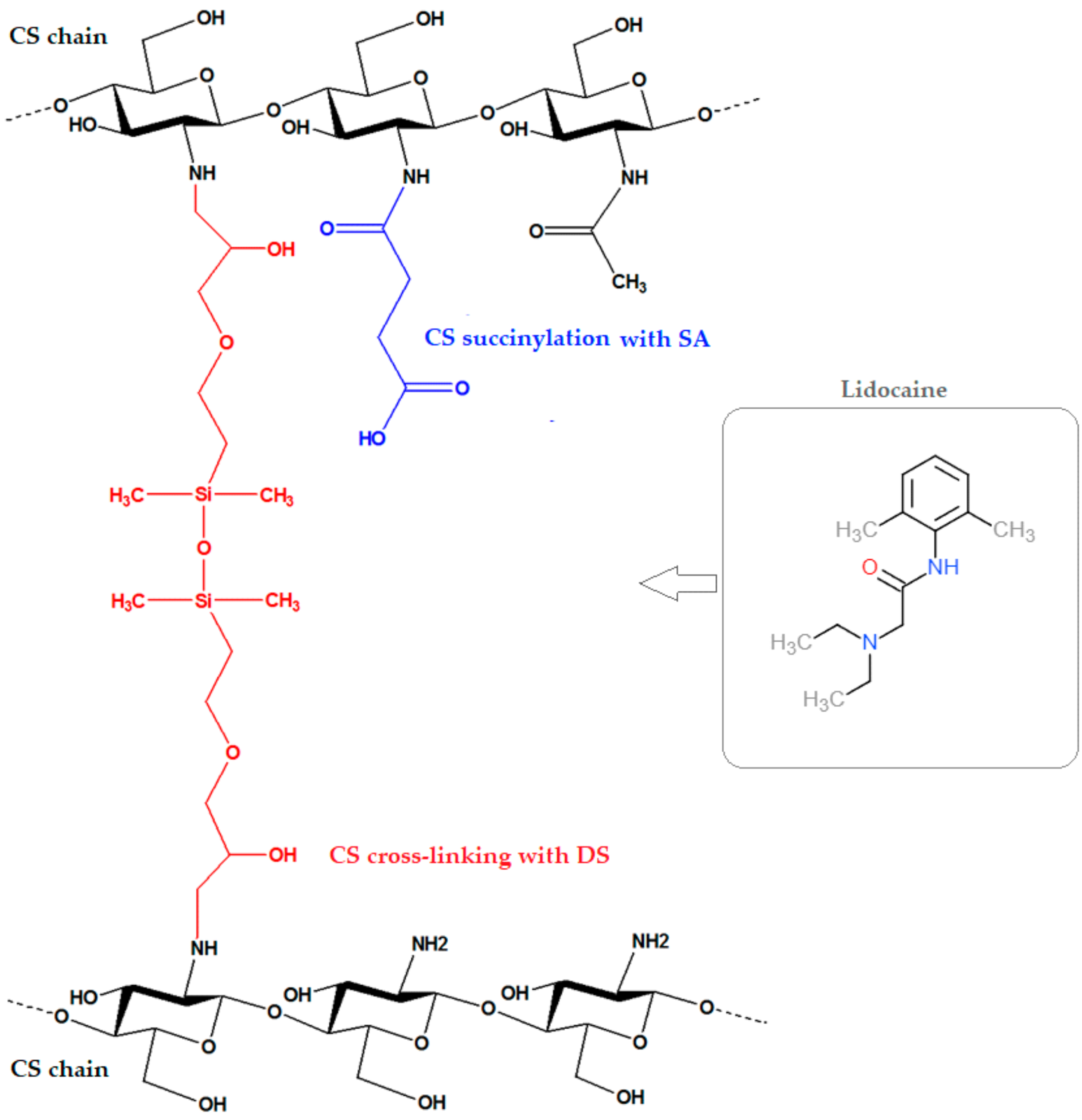

Figure S3. Schematic representation of the amphiphilic chitosan system (obtained by functionalization with succinic anhydride (SA) and cross-linking with 1,3-Bis(3-glycidyloxypropyl)tetramethyldisiloxane (DS)) for the embedment of insoluble lidocaine drug.

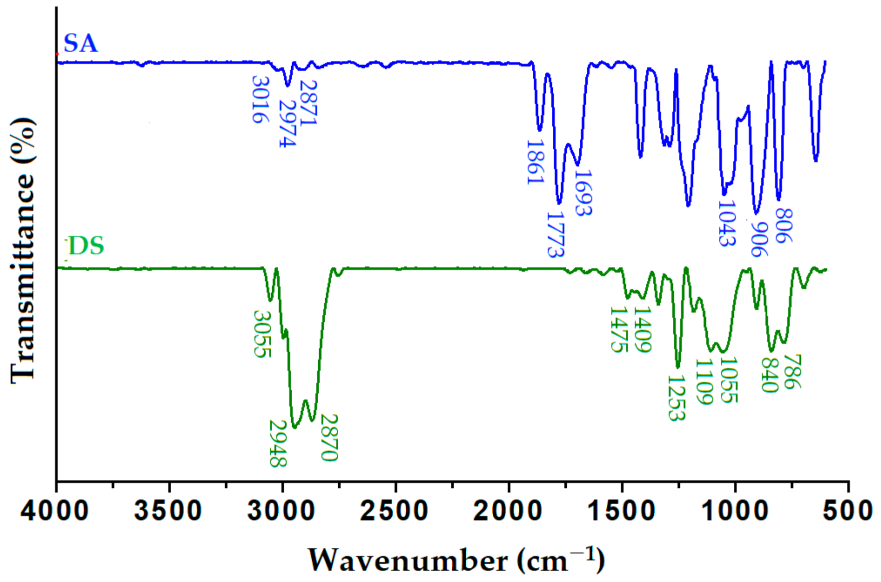

Figure S4. FTIR spectra of succinic anhydride (SA) and 1,3-bis-(3-glycidyloxypropyl) -1,1,3,3-tetramethyldisiloxane (DS).

In the characteristic spectrum of succinic anhydride, the main absorption bands are attributed as follows [36]:

- 2978 and 2871 cm^{-1} - asymmetric and symmetric C–H stretching vibrations;
- 3016 cm^{-1} - C–H vibrations specific to the AS cycle;
- 1863 cm^{-1} and 1773 cm^{-1} - symmetric and asymmetric stretching vibrations of the C=O group of anhydrides;
- 1689 cm^{-1} - symmetric stretching vibrations of the C=O group (succinic anhydride partially hydrolyzed);
- 1043 cm^{-1} and 906 cm^{-1} - C–O–C vibrations for succinic anhydride (five carbon atoms – membered).

The main absorption bands characteristic of DS are [36]:

- 3055 cm^{-1} - C–H stretching vibrations (epoxy group);
- 2945 cm^{-1} and 2837 cm^{-1} - C–H asymmetric and asymmetric stretching vibrations;
- 1254 cm^{-1} and 840-786 cm^{-1} - Si-CH_3 stretching vibrations;
- 1053-1109 cm^{-1} - Si–O–Si stretching vibrations and glycidyloxypropyl C–O–C unit.

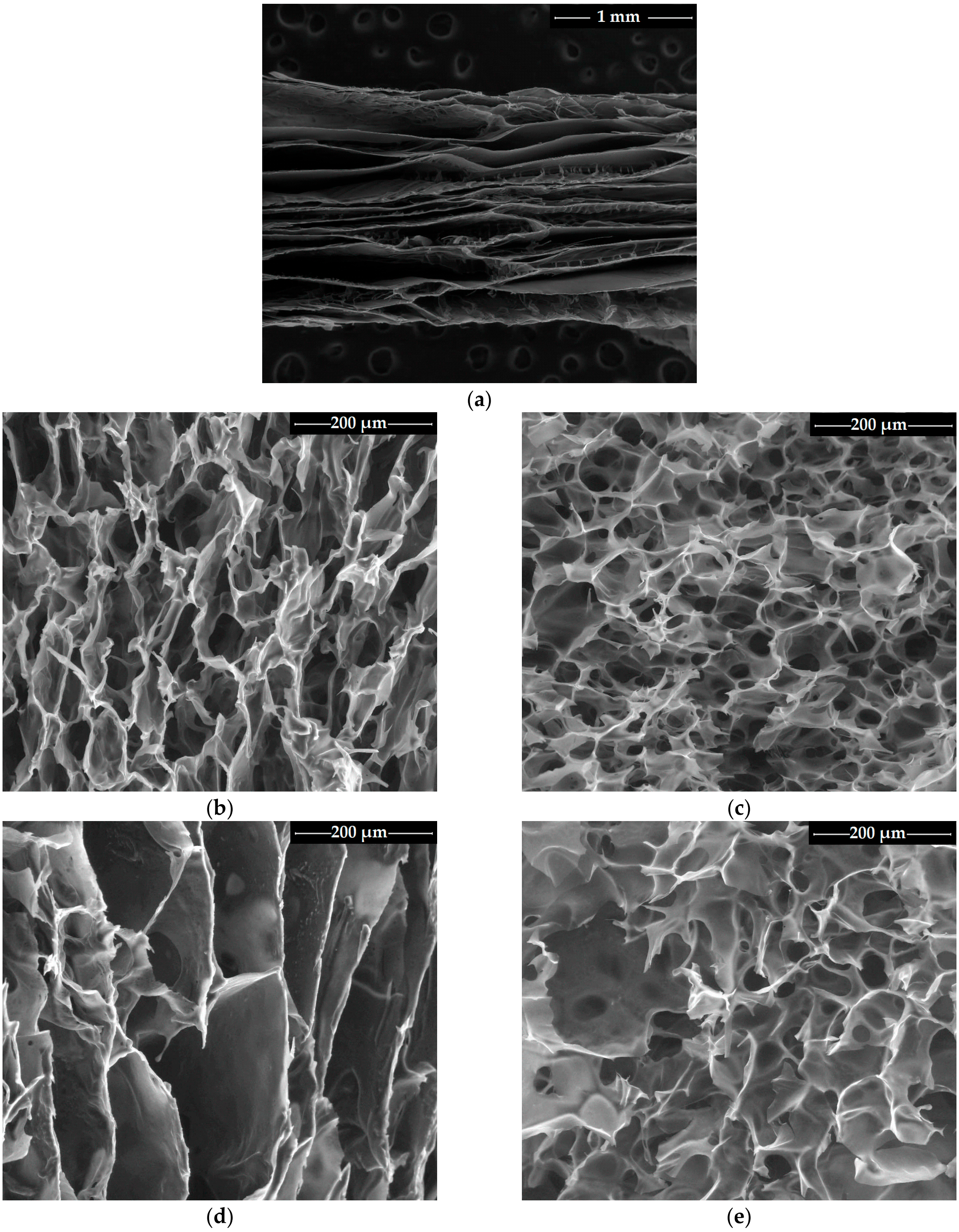

Figure S5. SEM images on the cross-section of non-charged chitosan membranes: (**a**) CS-A, (**b**) CS-SA/DS-1 and (**c**) CS-SA/DS-2, and of the lidocaine-charged membranes: (**d**) CS-SA/DS-1-LID and (**e**) CS-SA/DS-2-LID.

Element	Wt%	At%
CK	55.07	61.49
NK	07.72	07.39
OK	37.22	31.12
Matrix	Correction	ZAF

Element	Wt%	At%
CK	53.45	60.43
NK	07.69	07.45
OK	36.51	30.99
SiK	02.35	01.14
Matrix	Correction	ZAF

Element	Wt%	At%
CK	55.91	62.63
NK	07.57	07.27
OK	34.80	29.27
SiK	01.72	00.83
Matrix	Correction	ZAF

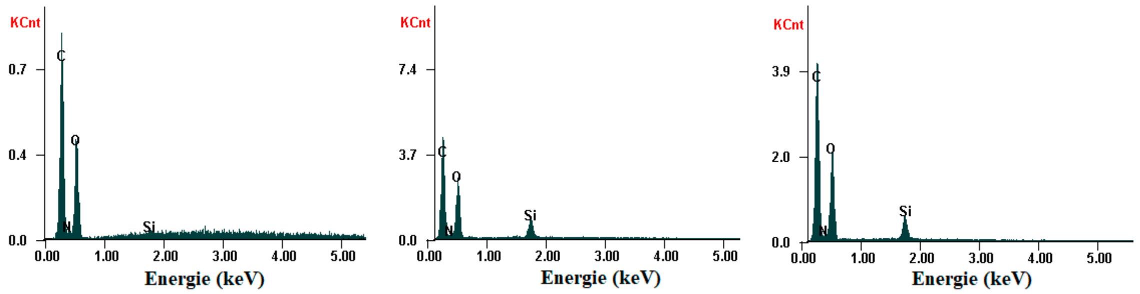

(**a**) (**b**) (**c**)

Figure S6. Structural elements determined by EDX in the section of: (a) CS-A unmodified membrane; (b) CS-SA/DS-1 modified membrane; and (c) CS-SA/DS-1-LID lidocaine-loaded chitosan membranes. Besides the specific C, O and N atoms of the chitosan and lidocaine compounds, the presence of Si atoms was noticed as the result of the cross-linking process.

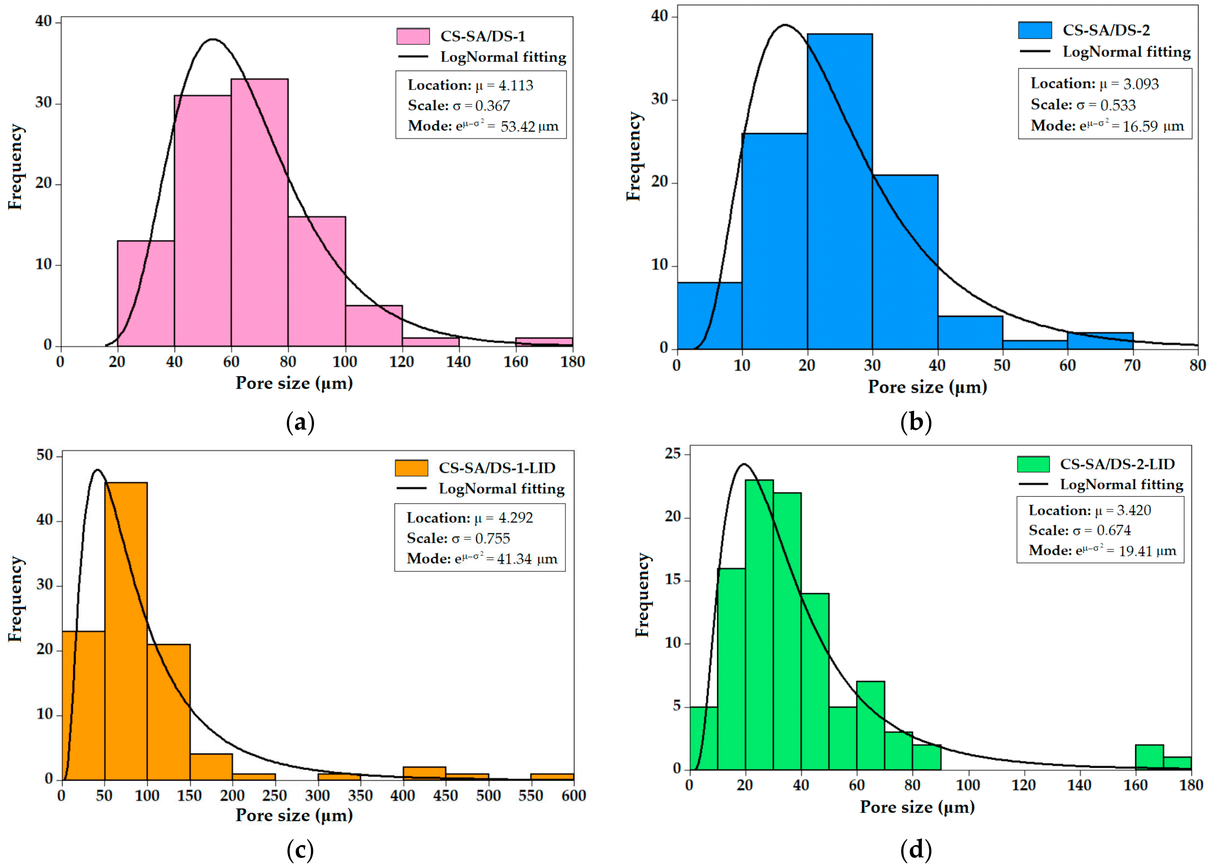

Figure S7. Histograms of pore distribution in the cross-section of: non-charged CS membranes (**a**) CS-SA/DS-1 and (**b**) CS-SA/DS-2; lidocaine-charged membranes (**c**) CS-SA/DS-1-LID and (**d**) CS-SA/DS-2-LID; the experimental data were fitted with LogNormal distribution function using Minitab 16 Software.

Table S1. Swelling kinetic models parameters (PSO - pseudo-second order; K-P - Korsmeyer-Peppas) and maximum swelling capacity values (g/g) of the porous membranes, after 5 h of immersion in PBS solution.

Film code	PSO model parameters	K-P model parameters	Swelling capacity after 5 h (g/g)
**CS-SA/DS-1**	$k_s = 0.60$<br>$S_{e1} = 11.35$<br>SD = 0.08	$k_p = 10.59$<br>n = 0.015<br>SD = 0.13	11.60
**CS-SA/DS-2**	$k_s = 1.50$<br>Se = 26.58<br>SD = 0.41	$k_p = 26.48$<br>$n = 9.32 \cdot 10^{-18}$<br>SD = 0.53	26.63
**CS-SA/DS-1-LID**	$k_s = 0.35$<br>Se = 8.63<br>SD = 0.41	$k_p = 7.85$<br>n = 0.018<br>SD = 0.53	8.60
**CS-SA/DS-2-LID**	$k_s = 2.51 \cdot 10^3$<br>Se = 24.81<br>SD = 0.41	$k_p = 23.80$<br>$n = 4.12 \cdot 10^{-18}$<br>SD = 0.53	22.80

[1] k_{s1} is the constants for the swelling rate, S_{e1} represents the theoretical swelling capacity at equilibrium, k_p is a polymeric network dependent constant and n is the diffusion exponential coefficient (SD = standard deviation for 10 data points).

Table S2. Release kinetic models (PFO - pseudo-first order and K-P - Korsmeyer-Peppas) parameters [1]. Lidocaine release efficiency (%) from chitosan porous membranes after 5 h.

Film code	PFO model parameters	K-P model parameters	Lidocaine release after 5 h (%)
**CS-SA/DS-1-LID**	k_r = 0.15<br>Sr = 85.16<br>SD = 1.66	k_{pr} = 32.14<br>n = 0.25<br>SD = 2.40	83.16
**CS-SA/DS-2-LID**	k_r = 0.30<br>Sr = 91.73<br>SD = 1.77	k_{pr} = 48.75<br>n = 0.17<br>SD = 2.41	95.24

[1] k_r - release constants; Sr - theoretical drug release, k_{pr} – K-P constant; n - diffusion coefficient (SD = standard deviation for 11 data (PFO) and 7 data la (K-P)).
